# Effectiveness and Implementation of Adapted Physical Activity Delivery Strategies for Older Adults Living With HIV in Ivory Coast: Protocol for a Type 2 Hybrid Randomized Controlled Trial

**DOI:** 10.2196/84677

**Published:** 2026-01-06

**Authors:** Nicolas Diadhiou, Nelly Assoumou, Joseph Tegbe, Herve Etchin Boa, Marie-Laura Bao, Simon Amador-Paz, Damien Vitiello, Patrick Coffie, Pierre Debeaudrap

**Affiliations:** 1 CEPED French National Research Institute for Sustainable Development Paris France; 2 ERL 1244 INSERM Paris France; 3 University Paris Sorbonne Paris France; 4 Université Paris Cité Paris, Île-de-France France; 5 PAC-CI program Treichville Teaching Hospital Abidjan Côte d'Ivoire; 6 FSU Com of Abobo Avocatier-Abidjan Abidjan Côte d'Ivoire; 7 Centre Medico-social El Rapha-Abidjan Abidjan Côte d'Ivoire; 8 ANRS-MIE (Emerging Infectious Diseases) Paris France; 9 URP 3625 Institut des Sciences du Sport Santé de Paris (I3SP), UFR STAPS Université Paris Cité Paris, Île-de-France France; 10 Dermatology and infectious diseases unit Félix Houphouët-Boigny University Abidjan Côte d'Ivoire

**Keywords:** Africa, aging, exercise, HIV, implementation science

## Abstract

**Background:**

With improved access to antiretroviral treatment, HIV infection has become a chronic disease, and the proportion of people living with HIV aged 50 years or older is increasing. However, the long-term evolution of this disease is associated with an increased risk of comorbidities and functional impairments, which negatively impact the social participation and quality of life of people living with HIV. In resource-limited countries, population aging is a new situation, and significant challenges remain unaddressed to respond to this demographic shift. Strong evidence supports the role of physical activity (PA) in improving health and decreasing functional limitations in many chronic conditions, including HIV. However, there is a lack of information on how to effectively implement this type of nondrug intervention in resource-limited contexts.

**Objective:**

This study aims to examine the effectiveness and implementation of 2 strategies to deliver an adapted PA program to older adults living with HIV.

**Methods:**

This is a prospective, randomized controlled trial following a type 2 hybrid design, with a dual focus on intervention effectiveness and implementation outcomes. It also includes prior formative research that provides information on the context and guides the implementation. Conducted in Ivory Coast, the study aims to randomize 180 people living with HIV aged 50 years or older, receiving antiretroviral treatment and presenting moderate functional limitations or disabilities into the following three arms: (1) a reference arm receiving a group-based PA program supervised by a coach, (2) an exploratory arm receiving a home-based PA program with remote supervision via phone calls and messaging apps, or (3) a control arm receiving health education sessions. The total follow-up period is planned for 12 months, with an initial 6-month active phase and then a 6-month maintenance phase. The primary effectiveness outcome is the increase in the number of steps in the 6-minute step-up test between baseline and 6 months. The secondary outcomes include changes in performance on other functional tests and improvements in cardiometabolic risk factors. The implementation outcomes include the acceptability, adoption, feasibility, and sustainability of the intervention.

**Results:**

This study is funded by ANRS Emerging Infectious Disease, which is also the study sponsor. It received ethical approval from the National Ethical Committee of Ivory Coast (00231 3124/MSHPCMU/CNESVS-km). As of manuscript submission, the baseline formative research has been completed, participants have been randomized, and they have started the PA activity program. Results dissemination will involve civil society and decision-makers through workshops and policy briefs.

**Conclusions:**

This study builds on previous research on healthy aging while living with HIV. Its hybrid design allows for a comprehensive evaluation of implementation processes and outcomes alongside effectiveness outcomes.

**Trial Registration:**

ClinicalTrial.gov NCT06139497; https://clinicaltrials.gov/study/NCT06139497

**International Registered Report Identifier (IRRID):**

DERR1-10.2196/84677

## Introduction

### Overview

With the scale-up of antiretroviral therapy (ART), the life expectancy of people living with HIV has improved dramatically over the last few decades, and HIV has become a chronic disease [1]. However, chronic HIV infection is associated with several long-term health challenges [2]. The chronic immune activation caused by HIV persistence, ART-related toxicity, and the occurrence of opportunistic infections increases the risk of cardiovascular diseases and musculoskeletal or neurocognitive disorders, among other conditions [3-8]. In resource-limited countries, the situation is compounded by factors such as malnutrition and opportunistic infections, which remain frequent and can increase the risk and severity of these chronic conditions [9,10]. These chronic HIV-related complications result in significant functional limitations, which translate into restrictions in daily activities and social participation, thereby negatively impacting the quality of life of people living with HIV [11-14]. According to available studies (mostly conducted in high-income countries), between one and three-quarters of people living with HIV on ART experience some form of activity limitation [15-20]. This wide range of reported prevalence likely reflects the variability of the study methodologies and population characteristics. When these limitations are ignored, they may compromise ART adherence and, consequently, treatment outcomes [14,16]. In addition, they are associated with depressive symptoms and financial hardship [19,20]. Addressing disability is therefore necessary to achieve the ambitious objective that most people living with HIV receive ART and maintain good health [21].

However, while more than two-thirds of people living with HIV reside in sub-Saharan Africa, there is a dearth of data documenting the situation of adults aging with HIV in these settings [22]. As life expectancy increases for both people living with HIV and the general population, the demographic profile of the HIV epidemic in sub-Saharan Africa is shifting, and an increasing proportion of people living with HIV are now older than 50 years [23]. Because of the significant differences in the characteristics of population with HIV (eg, sex distribution, treatment characteristics, genetics, and socioeconomic environment), data from high-income contexts cannot be simply extrapolated to African contexts.

### Physical Activity

There is now strong evidence that regular physical activity (PA) increases overall life expectancy [24] and enhances health outcomes in many chronic diseases [25], including HIV [26-34]. Various types of exercise are available and can be combined, such as aerobic exercise (low to moderate intensity), resistance exercise (strength and muscular resistance), flexibility exercise, and balance exercise [35]. Two systematic reviews including 30 randomized trials revealed that aerobic activity (with or without resistance) for at least 6 weeks significantly improved cardiorespiratory function, strength, weight, and body composition and improved quality of life [31,32]. When engaged in regularly, exercise training reduces the risk of disability caused by sarcopenia among people living with HIV [36], although studies on the long-term effects of exercise training are lacking. Some studies have reported a positive effect of PA on cognitive performance [37].

In a recent study conducted in Ivory Coast and including 300 adults living with HIV and 200 without infection, we found that, after the age of 50 years, people living with HIV who reported higher levels of PA achieved similar functional performance on the 6-minute walk test (6MWT) compared with controls [38]. In contrast, those who reported low PA had lower performance on the 6MWT [38].

### The Need for Research on How to Promote PA Among Aging Adults in Resource-Limited Contexts

Adopting a new lifestyle is a difficult task that requires motivation, opportunities, and capabilities. Therefore, the first step when implementing an intervention promoting PA is to obtain good knowledge of the participants’ living environment and of the contextual factors that may influence the adoption of the new behavior. Grounding interventions on one or more behavior change frameworks can increase their effectiveness and improve their transferability [39]. However, few interventions promoting PA have been implemented in African settings, and evidence on methods to achieve behavior change and successfully implement PA remains scarce.

The Health Action Process Approach (HAPA) is well-suited for developing activities directed toward the adoption and maintenance of PA [40-43]. The HAPA distinguishes the motivational and volitional phases during the behavior change process. The motivational phase is characterized by the building of motivation to be engaged in the new behavior, whereas the volitional phase relates to the engagement in and maintenance of the behavior. Furthermore, the HAPA identifies 6 constructs shaping the adoption process: intention, risk perception, outcome expectancies, self-efficacy, planning, and action control [40]. Among these, self-efficacy, planning, and action control are particularly influential in the volitional phase and have been shown to be more strongly associated with PA adoption and maintenance [41,43].

### The Need for an Implementation Strategy for PA Intervention

Despite strong evidence showing the effectiveness and cost-effectiveness of PA in preventing and reducing HIV- and age-related disabilities, very few programs have been implemented in sub-Saharan Africa, and none exist in West Africa [44]. Further research is needed to assess the effects of endurance and resistance training on older people living with HIV in African contexts [44]. Indeed, there is a lack of reference data on physical or neurocognitive outcomes in these populations, which are essential for establishing population norms and defining context-specific cutoffs. A significant gap persists between the scientific evidence produced by randomized clinical trials and its translation into actual and effective interventions [45]. Research is needed to explore how effective interventions can be integrated into real-world day-to-day activities of already busy health services, taking cultural differences into account [46].

In this context, this research aims to fill this gap by assessing the effectiveness and implementation of a structured PA program implemented through 2 strategies, group-based and home-based exercises, in improving the quality of life and exercise capacity of individuals aged 50 years and older living with HIV in Ivory Coast. It will primarily focus on the short-term effects of the intervention.

## Methods

### Study Design

**Figure 1:**
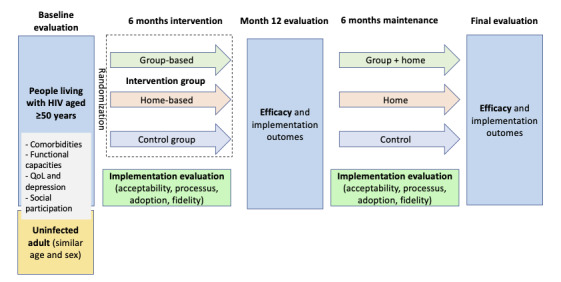
Study design. M12: month 12; QoL: quality of life.

VIRAGE+ (VIH Et Réadaptation Grâce À L’Exercice) is a prospective randomized controlled pilot trial with 2 phases: a 6-month active phase to implement the intervention and a 6-month maintenance phase to evaluate the sustainability of the intervention’s effects (Figure 1). This study adopts a type 2 hybrid design to assess both the effectiveness of the intervention on physical, cognitive, and cardiometabolic health outcomes and the implementation outcomes to identify factors influencing its adoption by users, as well as its integration into health care practices [47]. The trial comprises the following three arms: (1) a reference arm receiving a group-based PA program delivered by a certified coach, (2) an exploratory arm receiving a home-based PA program remotely delivered via mobile phone communication, and (3) a control arm receiving general advice on healthy behaviors. Participants in the control arm will be offered the intervention after the trial if it is proven effective. Details on the intervention are provided below.

This research is coordinated by the PAC-CI program in Ivory Coast and will be conducted in 2 health centers in Abobo, Ivory Coast.

### Study Population and Recruitment

The study population consists of people living with HIV aged ≥50 years, receiving ART for ≥12 months, residing in the catchment area of the study sites, and meeting at least one criterion for functional limitation or disability, as defined below. The exclusion criterion was any condition that could impede participation in the intervention. Eligibility criteria are listed in Textbox 1.

**Eligibility criteria.**

**Inclusion criteria**

- Age ≥50 years.
- Documented HIV infection.
- Stable antiretroviral therapy for ≥12 months.
- Residing in the Abobo district.
- The presence of at least one functional limitation or disability criterion among the following:
  - Performance in the 6-minute walk test ≤300 m
  - Handgrip strength ≤35 kg for men or ≤24 kg for women.
  - World Health Organization Disability Assessment Schedule (WHODAS) score ≥10.
  - Score at the short physical performance battery (SPPB) between 4 and 9.
  - Signed informed consent.

**Exclusion criteria**

- Medical contraindications to physical activity.
- Anticipated unavailability.
- Severe functional limitation (SPPB ≤3).
- Another health condition requiring priority care.

Recruitment will take place in 2 health centers in the Abobo district, a popular neighborhood on the outskirts of Abidjan. The first center, “Formation Urbaine Sanitaire Abobo Avocatier,” located in eastern Abobo, has an active cohort of approximately 1500 people living with HIV, including 500 individuals aged 50 years and older. The second center, “Centre Médicalisé Spécialisé El Rapha,” has an active cohort of 2000 people living with HIV, including approximately 650 individuals aged 50 years and older. Participants who fulfill the inclusion criteria are contacted by their clinicians, who will obtain their informed consent to participate in the study (Multimedia Appendix 1).

### Randomization and Blinding

The participants will be randomized using a computer-generated randomization list in a 1:1:1 ratio into one of three arms. Randomization will use fixed-size blocks and be stratified by study site. An independent team, not involved in intervention delivery, recruitment, or participant assessment, will generate and keep the randomization sequences. The arm allocation will be provided to investigators through a web application. An alternative procedure has been prepared in case of difficulties with the internet connection. Because of the nature of the intervention, participants and coaches will be aware of the arm allocated. However, the investigators responsible for the outcome evaluation are not involved in the intervention delivery and will remain blinded to the group assignments.

### Patient and Public Involvement

This research received valuable input from people living with HIV and health workers to refine the intervention design. A preliminary formative study was conducted to explore perceptions and social representations of aging and PA among the targeted population and to identify preferences for intervention delivery. Semistructured individual interviews (n=8) and focus groups (n=4) were conducted with potential participants, health workers, and coaches. This preliminary research revealed several key aspects of the intervention, including the composition of exercise groups (mixed- or single-gender), the acceptability and feasibility of home-based sessions, and practical considerations such as preferred schedules and methods for self-monitoring progress. These results were used to refine the design, framework, and materials of the intervention to ensure cultural appropriateness and feasibility.

In addition, a steering committee including people living with HIV, health workers, and representatives of the national AIDS program will follow the study progress and advise on its implementation and conduct, as well as on the result-sharing process.

### Study Intervention

The intervention consists of a 48-week exercise program including endurance, resistance, balance, and flexibility exercises performed 2-3 times per week. Endurance training sessions will include exercises conducted at 60%-70% of the maximal heart rate for at least 30 minutes, whereas resistance training sessions will involve exercises involving major muscle groups using resistance (eg, squats and lunges for lower limbs, and push-ups and dips for upper limbs) at 60%-70% of 1 repetition maximum, with 8-12 repetitions per set, performed for 2-4 sets. The Rating of Perceived Exhaustion (RPE) scale will also be used to calibrate the exercise intensity. The number of sets will gradually increase over 24 weeks to increase strength and endurance while minimizing the risk of injury. A session should last 60 minutes by the end of this period. Together, endurance and resistance training represent 80% of the activities proposed. Exercises are usually combined with circuit training, which involves performing a series of exercises in sequence with minimal rest to maximize efficiency and cardiovascular benefits. Alternatively, exercises can be performed in isolation, such as walking, to accommodate individual preferences and abilities.

### Program Initiation

The 2 intervention arms will start with a 3-week initiation phase designed to build trust between coaches and participants, ensure that the participants understand and are comfortable with the exercises planned, and establish a foundation for long-term adherence. This initiation phase includes weekly supervised sessions for both arms (group-based PA and home-based PA). The first supervised session will be devoted to an individual interview with the coach to assess participants’ motivation to engage in PA, set personalized goals (frequency, intensity, duration), and discuss the practical aspects of the PA program (ie, activity type, session format, visit schedule, and communication methods). Goals will be tailored on the basis of the initial physical assessments and the participants’ preferences. The following supervised sessions will focus on explaining the organization of a typical training session (warm-up, endurance, strength, and cooldown), how to use the RPE scale [48], and measure the heart rate, teaching safety aspects of physical training, and discussing the integration of the PA exercises into daily routines when performed at home.

### Delivery Strategies

A total of 2 delivery approaches for the PA program are proposed. In the group-based arm, participants will take part in group sessions directly supervised by a coach. The supervised sessions are planned once a week during the first 24 weeks (active phase) and transition to once a month during the 24-week maintenance phase. This construction in 2 phases is based on behavior change frameworks such as the HAPA or the transtheoretical model of change, which distinguish 3 components (initiation, maintenance, and recovery) in the process of adoption of a new behavior [40]. The other session of the PA program will be performed at home, and the overall weekly PA will be reviewed with the coach at the end of the supervised session.

In the home-based arm, participants will perform the same exercise program at home exclusively. They will be called every week by a research assistant to review the activities performed and collect information on their adherence and perceptions of the program. During this call, the research assistant will also provide positive feedback on the participants’ activity, help them address challenges, and reinforce their motivation. The weekly program will be shared via phone calls and sent via SMS text message or WhatsApp according to participants’ preferences. Participants can access and download videos demonstrating each exercise, as well as a training book displaying all exercises they will have to perform during the program.

Various components of the HAPA model are targeted in both interventional arms, even though the actual activities will differ between the arms. In each interventional arm, participants will receive motivational interviews with a coach or a research assistant [49,50]. Motivational interviews will initially cover the advantages of PA and the disadvantages of sedentarism and discuss participants’ intentions to engage in the adapted PA program. They will avoid a paternalistic or authoritative approach and help to build commitment to adopt this new behavior by discussing an action plan with clear, realistic goals and offering optimistic views. Motivational interviews will continue during the intervention, either through face-to-face discussions with the coach or through phone calls. The focus of these interviews will progressively shift toward self-monitoring and action planning. The participants will be asked to monitor their PA on a weekly basis using a dedicated instrument. During the interviews, they will review their goals as well as the potential problems faced and solutions identified, and they will be able to adapt their program to their goals with the help of the coach or research assistant. When needed, coping strategies will be explored and discussed.

### Maintenance Phase

In the maintenance phase, participants will be encouraged to maintain their exercise routine, with 3 sessions per week. The frequency of supervised group sessions will gradually decrease, and similarly, the home-based group will experience a gradual reduction in follow-up call frequency. This gradual reduction is intended to support the development of self-efficacy and long-term adherence to the exercise regimen.

### Control Group

Participants in the control group will receive monthly 30- to 60-minute health education sessions. This frequency ensures regular engagement without overburdening participants. These sessions will promote scientifically validated lifestyle habits for healthy aging, including balanced diet recommendations, age-appropriate advice for PA, and strategies for daily PA integration. The control arm will be offered the intervention after the trial if it is proven effective.

### Effectiveness Outcome Assessment

In this type 2 hybrid design, both effectiveness and implementation will be evaluated and receive equal attention. For clarity, effectiveness outcomes are presented in this section, and implementation research is presented in the following section. The primary effectiveness outcome is the difference in the number of steps completed during a 6-minute step-up test (6MST) at 6 months. The 6MST is a submaximal aerobic test that provides information on cardiorespiratory and functional capacities. Although less accurate than maximal exercise tests (with ergocycles or treadmills), the 6MST was selected because it does not require sophisticated technology and is easier to implement in resource-limited settings. Compared with the 6MWT, a reference for submaximal exercise tests [51], the 6MWT has similar good reproducibility and reliability [51,52] but is easier to perform. In addition, it is slightly more demanding, which is appealing for the evaluation of adults with relatively good health. The other effectiveness outcomes included in this study reflect key health outcomes relevant to aging individuals living with HIV and will be measured at 6 and 12 months. These include evaluations of strength, balance, cognitive performance, quality of life, depression, daily autonomy, social participation, blood pressure, and glucose metabolism. Details on the operationalization of all effectiveness outcomes are provided in Multimedia Appendix 2. All effectiveness outcomes will be assessed by investigators blinded to the participants’ assigned intervention arm (single-blind).

### Implementation Research

The implementation research aims to evaluate the success of program implementation and to determine which factors influence it and how. The implementation outcomes will be assessed using the RE-AIM (Reach, Efficacy, Adoption, Implementation, and Maintenance) [53] evaluation framework and cover the reach, adoption, effectiveness, satisfaction, resource requirements, and sustainability aspects of implementation. The RE-AIM framework was selected for its comprehensive approach, which evaluates both individual- and organizational-level outcomes critical for public health interventions. A description of these outcomes and of the measurement methods is provided in Table 1. The analysis of the implementation determinants will examine different levels of factors that may influence one or several aspects of the implementation process and will be guided by the Consolidated Framework of Implementation Research (CFIR) [54]. It starts with the contextual factors, which include the characteristics of the 2 implementation sites and the organizational aspects of the health units and fitness centers involved. Elements of internal context of interest are the implementation climate, the perception of PA by health workers and users, the attitudes toward change, the values and norms related to the intervention, the attitudes of the coaches toward people living with HIV and older people, and their confidence in their ability to conduct the program.

**Table 1:** RE-AIM (Reach, Efficacy, Adoption, Implementation, and Maintenance) framework outcomes.

Dimension and variables			Measurement method
**Reach**			
	Number of participants who did not consent to participate and number not included in the trial	Monitoring (registers)	
	Reasons	Interviews and registers	
	Number lost to follow-up	Monitoring (baseline to month 6)	
**Efficacy**			
	Proportion of participants who have ≥1 session per week	Monitoring (registers and journals)	
	Proportion with participation of ≥75% of the sessions	Monitoring (registers and journals)	
	Reason for adherence and nonadherence	Interviews	
**Adoption**			
	Acceptability	Questionnaires (weeks 12, 24, and 48) and interviews	
	Satisfaction	Interviews	
**Implementation (fidelity)**			
	Number of modifications implemented	Monitoring and observations	
**Maintenance (phase 2)**			
	Proportion of participants with participation of ≥75% of sessions	Monitoring (≥6 months)	
	Proportion of participants with decreasing participation	Monitoring (≥6 months)	
	Proportion of participants having ≥1 session per week	Monitoring (≥6 months)	
	Reasons	Interviews	

The acceptability of the PA program will be assessed before and during implementation using the theoretical framework of acceptability [55], which encompasses the following constructs: affective attitude, burden, perceived effectiveness, ethicality, intervention coherence, opportunity costs, and self-efficacy.

The implementation outcomes and determinants will be measured using quantitative methods (process indicators and questionnaires) along with qualitative methods (semistructured interviews with participants and observations). Both approaches will be implemented concurrently with systematic triangulation of data as the project progresses.

### Evaluations and Data Collection

Data on the intervention’s efficacy and its implementation will be collected at multiple time points during the study to address the 2 main objectives of this research.

#### Initial (Baseline) Evaluation

This evaluation follows the International Classification of Functioning, Disability, and Health (ICF) framework [56], chosen for its holistic approach to health, encompassing physical, functional, and social dimensions. It provides information on participants’ physical capacities (strength, balance, and endurance), functional limitations, and social participation. In addition, cardiometabolic risk will be assessed via clinical (anthropometric characteristics and blood pressure) and biological (fasting glucose, glycated hemoglobin, cholesterol levels, and urinary branched-chain amino acid detection) evaluations. Urinary branched-chain amino acid is a screening test for insulin resistance, and elevated fasting glucose or glycated hemoglobin indicates impaired glucose metabolism and type 2 diabetes mellitus. Obesity, type 2 diabetes, insulin resistance, high blood pressure, and dyslipidemia are frequent conditions among people aging with HIV; these conditions are associated with major health challenges and can be effectively prevented through PA. Finally, depressive symptoms, dietary quality, alcohol consumption, and usual PA levels will be evaluated using validated questionnaires. The list of the evaluations performed is presented in Table 2, and a description of the physical tests is provided in Multimedia Appendix 2.

**Table 2:** Summary of the evaluations performed (a detailed description of the tests is provided in Multimedia Appendix 2).

	Initial	Week 12	Week 24	Week 48
Clinical examination	✓		✓	✓
Heart rate, blood pressure, height, weight, body composition	✓		✓	✓
Sociodemographic characteristics and medical history	✓			
Diet quality questionnaire				
Functional test: 6-minute step-up test, grip strength measurement, gait speed test, stand-up test	✓		✓	✓
Balance tests (Y balance test, tandem, semitandem)	✓		✓	✓
Glycated hemoglobin, fasting blood glucose, cholesterol LDL^a^ and HDL^b^, triglyceride levels	✓			✓
IDIR^c^	✓		✓	✓
Neurocognitive evaluation (Neuroscreen)	✓			✓
Self-reported PA^d^ (GPAQ^e^ or IPAQ^f^)	✓		✓	✓
Social participation and activity limitation: IADL^g^, WHODAS-2^h^, HDQ^i^	✓		✓	✓
Quality of life (WHOQoL^j^-short)	✓		✓	✓
Alcohol consumption (AUDIT^k^)	✓			
Depression (PHQ-9^l^)	✓		✓	✓
Satisfaction with PA (questionnaire)		✓	✓	✓
Acceptability of the PA program		✓	✓	✓

^a^LDL: low-density lipoprotein (cholesterol).

^b^HDL: high-density lipoprotein (cholesterol).

^c^IDIR: urinary branched-chain amino acid measurement to detect insulin resistance.

^d^PA: physical activity.

^e^GPAQ: Global Physical Activity Questionnaire.

^f^IPAQ: International Physical Activity Questionnaire.

^g^IADL: Instrumental Activities of Daily Living.

^h^WHODAS-2: World Health Organization Disability Assessment Schedule-2.

^i^HDQ: HIV Disability Questionnaire.

^j^WHOQoL: World Health Organization Quality of Life.

^k^AUDIT: Alcohol Use Disorders Test.

^l^PHQ-9: Patient Health Questionnaire 9.

#### Follow-Up Visit (Week 12)

The aim of this visit is to identify potential challenges faced by participants in the intervention groups, particularly those in the home-based group. It will involve a structured interview with the coach to discuss the training experiences and potential problems encountered, and questionnaires on self-reported PA (Internation Physical Activity Questionnaire), satisfaction with PA, and acceptability of the intervention. The interviews will explore barriers to adherence, perceptions of exercise intensity, and suggestions for program improvement. The major issues identified will lead to tailored adjustments, which will be documented and monitored for their effects.

Some participants will also have semistructured in-depth interviews with a social scientist to delve deeper into their experiences during the initial months of the intervention.

#### Intervention (Week 24) and Maintenance (Week 48) Evaluation Visits

These visits will provide data for assessing the primary and secondary effectiveness outcomes (Table 2). The same evaluations as for the baseline will be performed alongside questionnaires on satisfaction, acceptability, and quality of life. In addition, in-depth semistructured interviews will be conducted with a subset of participants.

#### Data Management

Data will be collected on paper forms before being entered into a web application designed for this study. It includes an application server and database servers hosted at the PAC-CI program in Ivory Coast with regular backup on a second server located in another site and secure access via https, passwords, and multiple firewalls. The data entered into the database are monitored with consistency checks. In addition, a monitoring of the data collected is performed by the clinical research assistant according to a monitoring plan.

### Statistical Analysis

#### Sample Size

In a previous study, participants aged 50 years or older with HIV who reported high PA presented an improvement of approximately 10% in performance compared with those with low activity in the 6MWT (422 meters versus 380 meters) or the 6MST test (115 steps vs 105 steps) [38]. This difference is consistent with an estimated minimal clinically important difference of about 40 meters reported in other studies on older people or people with chronic disease [57,58], which translates into a difference of approximately 10 steps. Using these results, 50 participants per group will ensure 80% power to detect a significant difference between the intervention and control groups, assuming a type I error rate of 0.025 (adjusted for multiple comparisons regarding the primary end point). To accommodate attrition, each planned group size was increased by 20%, resulting in a total planned sample size of 180 participants. Importantly, this pilot study is powered to compare both intervention arms with the control arm but not to test for noninferiority between the exploratory and reference arms.

#### Data Analysis

Primary analyses will use the intention-to-treat approach, considering all included participants in their initial groups and using all data, regardless of the intervention participation level. If protocol deviations are frequent, a per-protocol analysis will be conducted, with a focus on participants who followed the intervention.

The primary outcome analysis will compare scores between groups with modified covariance analysis (ANCOVA), using a linear regression model to analyze postintervention results in relation to the intervention group while accounting for baseline values, age, and sex. Robust linear regression will be used if necessary. A parallel gatekeeping procedure with the following four families of end points will be used to control the type I error risk for secondary end points: (1) the primary family includes the end points related to physical capacity (6MST, strength, and 30-second stand-up test), (2) comorbidities (high blood pressure, high fasting glycemia, obesity, and insulin resistance), (3) disability (HIV Disability Questionnaire scores), (4) mental health (quality of life and Personal Health Questionnaire 9 scores). End points of comorbidities, disability, and mental health families will be analyzed only if at least one primary end point is significant (with the Hommel method used to control for type I error). Secondary end points will be analyzed using the same approach with the ANCOVA model. Exploratory analyses will analyze repeated data using mixed-effects linear models, and Generalized Additive Mixed Model will be used to model nonlinear relationships. Missing data will be described and imputed using multiple imputations by chained equations, assuming that they are randomly missing, with estimates obtained via Rubin’s method.

### Ethical Considerations

This study is carried out in compliance with the ethical principles set out in the current revised version of the Declaration of Helsinki and with the International Council for Harmonisation guidelines. It received ethical approval from the National Ethical Committee of Ivory Coast (number 00231 3124/MSHPCMU/CNESVS-km issued on October 11, 2024). It is registered on the ClinicalTrials.gov website (NCT06139497). Changes to the study protocol should be validated by the sponsor and scientific committee and will be promptly communicated to the investigators and ethics committee. The study has been notified to and obtained authorization from the French data protection authority for the data processing carried out as part of this research.

A knowledge transfer plan will be prepared. The study results will be published in journal articles and presented at relevant scientific conferences. Results dissemination will also involve civil society and decision-makers through workshops and policy briefs.

Because of the limited risk of this nontherapeutic trial, there is no data monitoring committee. However, a scientific committee meets at least once a year to provide guidance on the study rationale and progress. Adverse events will be reported via specific forms to the sponsor.

## Results

This study is funded by ANRS Emerging Infectious Disease (ANRS-MIE), which is also the study sponsor. The trial has received approval from the National Ethical Committee of Ivory Coast and from the French Data Protection Authority (CNIL, DR-2024-229). As of manuscript submission, the baseline formative research has been completed, and all participants have been recruited and randomized. The PA program has started with an overall participation rate of 85% (102/120) though some participants started the program with several months delay. The 6-month evaluation will start in November 2025, and the study is expected to be completed in June 2026.

## Discussion

### Overview

This study protocol outlines the design and methodology of the VIRAGE+ trial, a multicenter, randomized controlled pilot study aimed at evaluating the effectiveness and implementation of a structured PA intervention for people living with HIV aged 50 years and older in Ivory Coast. This trial addresses the need to better understand how PA can mitigate age-related and HIV-associated disabilities in resource-limited settings and improve the exercise capacity and quality of life of older people living with HIV.

### Contribution to Existing Knowledge and Strengths

Although evidence is accumulating on the beneficial effects of PA on the physical and mental health of people living with HIV, most studies have been conducted in high-income countries with limited applicability to African contexts. Moreover, socioeconomic and cultural factors affect the risk of HIV and age-related functional limitations as well as the risk of chronic disease, as shown by the significant heterogeneity in the prevalence of comorbidities and functional limitations among people living with HIV found in studies originating from sub-Saharan Africa. These contextual factors are therefore important to consider when designing and implementing preventive interventions. The mixed methods approach of this study, along with its hybrid design and preliminary formative research, will help gather information on different aspects of the context (eg, cultural, socioeconomic, and psychological characteristics).

Another important goal of this research is to investigate how a program of adapted PA could be embedded into routine HIV health care in a resource-limited context. To achieve this goal, a 3-arm hybrid trial was designed with a dual purpose. First, the group-based arm (reference strategy) will provide information on the scale of change that might be achieved with a structured PA program in the specific context of urban HIV health care centers in Ivory Coast. The home-based arm will provide evidence on the effectiveness and implementation of a simplified intervention, which is more feasible for adoption by stakeholders. An important strength of this research is the type 2 hybrid design, which will help to identify factors influencing the adoption and maintenance of PA together with evaluating their effectiveness. Various methods will contribute to implementation research, encompassing process evaluation, observations, and interviews with participants and stakeholders. The information collected will be triangulated and discussed with people living with HIV, health care workers, and program managers. Importantly, intervention implementation and implementation evaluation will be guided by frameworks [59]. The use of one or several frameworks should be considered a heuristic approach, facilitating analysis, organization, and knowledge sharing.

The intervention leverages the HAPA framework to enhance adherence and sustain long-term behavioral change. This framework was selected on the basis of preliminary formative research and helped organize the intervention’s logic model. By targeting constructs such as self-efficacy, risk perception, and planning, the program is designed to address common challenges faced by aging people living with HIV, including a lack of confidence in their ability to engage in regular PA and competing socioeconomic priorities. Other factors may emerge during program implementation, and alternative frameworks could be identified through empirical data. Additional frameworks were selected to guide the implementation research [60]. The RE-AIM framework was selected to assess the different aspects of implementation success. Because it is a widely used and comprehensive framework, this approach facilitates the dissemination of the study results. The implementation research also aimed to identify factors that influence implementation using the CFIR. This framework was selected because it provides a general and systematic approach to the evaluation of implementation determinants and can easily be translated into interview guides and frameworks for questionnaire and indicator collection.

### Anticipated Challenges and Mitigation Strategies

#### Adherence or Attrition

Adherence to PA interventions is a challenge [61], particularly in resource-limited settings where socioeconomic factors and competing priorities may diminish participation [62]. To address this, this study will incorporate motivational interviews, personalized goal setting, and regular follow-ups, which have been shown to increase self-efficacy and thereby enhance engagement [63]. Additionally, the home-based arm will include flexible scheduling and remote support via phone calls and messaging apps, accommodating participants with time constraints or mobility limitations.

#### Contextual Factors and Participatory Approach

Cultural perceptions of aging, PA, and HIV may influence participants’ willingness to engage in the intervention. For example, group sessions are expected to foster social support, which has been shown to promote PA maintenance among older people [61], whereas home-based exercises can offer privacy for individuals who may feel stigmatized, a critical challenge among people living with HIV [64-66]. The formative research conducted during the design phase of the study provided information on the perceptions of the targeted population of the program, gathered suggestions on its implementation, and helped to identify potential implementation barriers and facilitators. Therefore, it allows for culturally tailored intervention materials and strategies. This iterative feedback from key stakeholders is critical for refining and adapting the intervention and ensuring its relevance [67]. Although this research is not strictly participatory [68], people living with HIV, health workers, and coaches will be consulted before and during the study and will be invited to discuss different aspects of the intervention, study design, and results. We believe that the discussions will be enriched as the study progresses, insofar as the stakeholders will become more familiar with the intervention and the research process, strengthening the confidence bound between them and the researchers.

#### Broader Implications

The situation of older people in resource-limited countries requires particular attention because of the unique challenges posed by resource limitations. Access to health care in many resource-limited countries is limited, especially for aging populations, and noncommunicable diseases are rarely screened for. However, HIV care offers a unique opportunity for intervention, as ART programs often provide free or subsidized services. These programs could serve as entry points for integrating PA into routine care. In addition, some people living with HIV may already benefit from preventive health care services, such as cervical cancer screening, highlighting the potential for leveraging existing health care infrastructure to introduce scalable PA interventions.

### Limitations and Perspectives

As a pilot study, VIRAGE+ is not powerful enough to detect noninferiority between the 2 intervention arms, which limits the conclusions that can be drawn about their relative effectiveness. However, these findings will inform the design of larger, more definitive trials. Additionally, the study relies on self-reported measures for certain outcomes, such as the PA performed, which may introduce reporting biases. Future research should explore the use of objective monitoring tools, such as wearable activity trackers, to complement self-reported data.

Another limitation is the study’s focus on urban health centers. Expanding similar interventions to rural areas in future studies will be critical to ensuring equitable access to PA programs for all people living with HIV.

### Conclusion

The VIRAGE+ trial represents a step toward addressing the dual challenges of aging with HIV in resource-limited settings, such as in Ivory Coast. By combining the evaluation of health outcomes with a detailed assessment of implementation processes, this study aims to generate actionable data that can inform the integration of PA into HIV care. The findings are expected to contribute to improving the quality of life of aging people living with HIV in Ivory Coast and serve as a protocol for similar interventions using adapted PA in other low- and middle-income countries.

## Appendix

Multimedia Appendix 1 Informed consent form.

Multimedia Appendix 2 Description of the physical tests performed.

Multimedia Appendix 3 Peer review comments from the l'Agence nationale de recherches sur le sida et les hépatites virales - Maladies infectieuses émergentes (National Research Agency for HIV, viral hepatitis and emerging infectious diseases; ANRS-MIE, France).

## Abbreviations

6MST: 6-minute step-up test
6MWT: 6-minute walk test
ANCOVA: covariance analysis
ANRS-MIE: ANRS Emerging Infectious Disease
ART: antiretroviral therapy
CFIR: Consolidated Framework of Implementation Research
HAPA: Health Action Process Approach
ICF: International Classification of Functioning, Disability and Health
PA: physical activity
RE-AIM: Reach, Efficacy, Adoption, Implementation, and Maintenance
RPE: Rating of Perceived Exhaustion
VIRAGE+: VIH Et Réadaptation Grâce À L’Exercice
