# Supplementary material for: Effectiveness and Implementation of Adapted Physical Activity Delivery Strategies for Older Adults Living With HIV in Ivory Coast: Protocol for a Type 2 Hybrid Randomized Controlled Trial
Source: JMIR Res Protoc. 2026 Jan 6;15:e84677. doi: 10.2196/84677 (PMC12820546; doi:10.2196/84677)
Supplement: Multimedia Appendix 1 [file resprot_v15i1e84677_app1.pdf]

Faisabilité et efficacité d'un programme pilote d'activité physique sur les limitations fonctionnelles et le handicap associés au VIH chez les plus de 50 ans en Côte d'Ivoire (étude VIRAGE+ ANRS 0396)

FORMULAIRE DE CONSENTEMENT DES PARTICIPANTS
Version 2.0 du __/__/2024
ayant reçu l'avis favorable
du Comité d'éthique de Côte d'Ivoire le __/__/2024

Promoteur : Inserm-ANRS Maladies Infectieuses Emergentes
Institut national de la santé et de la recherche médicale (Inserm)
ANRS Maladies Infectieuses Emergentes (ANRS | MIE)
2, rue d'Oradour sur Glane
75015 Paris
Tél. : 01 53 94 60 00 - Fax : 01 53 94 60 01

Responsables scientifiques de l'étude :
- Dr. Patrick Coffie (PAC-CI, Abidjan, Côte d'Ivoire)
- Dr. Pierre Debeaudrap (CEPED, Paris, France)

Mme, M____*(nom et prénom)*

Je certifie :

- Avoir reçu la note d'information du xx/xx/xxxx,
- Avoir eu la possibilité de poser toutes les questions que je souhaitais sur la nature, les objectifs, les risques potentiels et les contraintes liés à ma participation à cette recherche,
- Avoir eu un délai de réflexion suffisant entre l'information et le consentement.

J'ai compris les contraintes, les risques potentiels et les bénéfices liés à ma participation à cette recherche qui durera quelques heures.

J'ai compris que je suis libre d'interrompre ma participation à cette recherche à tout moment sans avoir à expliquer pourquoi mais je ferai mon possible pour en informer le médecin qui me suit. Cela ne remettra pas en cause la qualité de mes soins ultérieurs. J'ai eu l'assurance que les décisions qui s'imposeront pour ma santé seront prises à tout moment conformément à l'état des connaissances sur le VIH.

J'accepte que les données enregistrées à l'occasion de cette recherche soient collectées, traitées et informatisées. J'ai bien compris que le droit d'accès prévu par la loi française du 6 janvier 1978 modifiée relative à l'informatique, aux fichiers et aux libertés et le Règlement Général sur la Protection des Données (RGPD - Règlement (UE) 2016/679) s'impose à tout moment au médecin qui me suit dans le cadre de la recherche et que je pourrai exercer mes droits de rectification, d'effacement, d'opposition et à la limitation.

J'accepte que les scientifiques impliqués dans cette recherche ainsi que les personnes mandatées par le promoteur, les autorités de santé en Côte d'Ivoire et à l'étranger aient accès aux informations me concernant dans le respect le plus strict de la confidentialité.

Mon consentement ne décharge en rien les organisateurs de la recherche de leurs responsabilités. Je conserve tous les droits garantis par la loi.

A l'issue de la recherche, **je pourrai être informé(e)** des résultats globaux par l'intermédiaire du médecin de la recherche.

J'ai bien été informé(e) que mes données pourront être utilisés pour des recherches ultérieures à des fins scientifiques sur le VIH sauf si je m'y oppose.

J'ai bien été informé(e) de la fixation, la reproduction sur support d'enregistrement de ma voix dans le cadre de cette recherche, ainsi que de l'utilisation qui en sera faite

**J'accepte librement de participer à cette recherche dans les conditions précisées dans la note d'information.**	☐ **OUI** ☐ **NON**
**J'accepte la conservation de mes données pour des recherches ultérieures sur le VIH**	☐ **OUI** ☐ **NON**
**J'accepte la conservation de mes prélèvements biologiques pour des recherches ultérieures sur le VIH et le vieillissement (Si et seulement si ceux-ci ne permettent pas de m'identifier; s'ils le font, un consentement éclairé spécifique me sera demandé).**	☐ **OUI** ☐ **NON**
**J'accepte de participer à des entretiens approfondis avec une sociologue et que cet entretien soit enregistré par un dictaphone**	☐ **OUI** ☐ **NON**
**J'accepte d'être recontacté pour un suivi après la fin de l'intervention**	☐ **OUI** ☐ **NON**

Le |__|__| |__|__| |__|__|__|__|	**Signature du Participant :**
Nom, Prénom:<br>Le |__|__| |__|__| |__|__|__|__|	**Signature de la Personne de confiance :**

Je soussigné(e), <Dr>____certifie avoir communiqué au participant toute l'information relative à cette recherche, avoir répondu à ses questions et recueilli son consentement.

Le |__|__| |__|__| |__|__|__|__| Nom du service :<br>Adresse :<br>Téléphone :	**Signature du <Médecin> :**
