# Supplementary material for: Effectiveness and Implementation of Adapted Physical Activity Delivery Strategies for Older Adults Living With HIV in Ivory Coast: Protocol for a Type 2 Hybrid Randomized Controlled Trial
Source: JMIR Res Protoc. 2026 Jan 6;15:e84677. doi: 10.2196/84677 (PMC12820546; doi:10.2196/84677)
Supplement: Multimedia Appendix 2 [file resprot_v15i1e84677_app2.pdf]

Supplementary material

Test	Description	Metric	Timing
6-minute step-up test	Number of step up and down cycles performed over a 6-minute period with a 15 cm step	Count	Inclusion, M6, M12
6-minute walk test	Distance covered during 6 minutes	Total distance measured during back-and-forth walking over a 20 m distance	Inclusion, M6, M12
Grip strength	Measures hand grip strength in kg (2 tests for each arm)	Dynamometer JAMAR	Inclusion, M6, M12
5 times stand up and sit	Time taken to stand up and sit down 5 times	Stopwatch	Inclusion, M6, M12
Y balance test	Distance reached in cm by each foot (3 trials per foot) in three directions: anterior, posteromedial, posterolateral	Average reach distance in each direction (cm) per foot	Inclusion, M6, M12
Short Physical Performance Battery			Inclusion, M6, M12
Blood pressure	Measured at rest in a supine position for at least 10 minutes (without movement)	Blood pressure cuff	Inclusion, M6, M12

Disability		WHODAS	Inclusion, M12
Quality of life scale	Questions with a Likert-type scale	WHOQol - Bref	Inclusion, M6, M12
IADL (Instrumental Activities of Daily Living)	Questions with a Likert-type scale	DPI	Inclusion, M6, M12
HDQ	Questions with a Likert-type scale	HDQ	Inclusion, M6, M12
Depression	Questions with a Likert-type scale	PHQ-9	Inclusion, M6, M12
Physical activity	Questions with a Likert-type scale	GPAQ	Inclusion, M6, M12
Satisfaction	Questions with a Likert-type scale	Perceived enjoyment of physical activity	S12, S24, S48
Acceptability	Questions with a Likert-type scale	Four dimensions of acceptability (perceived effectiveness, relevance, convenience, and perceived risk) assessed using a 5-point scale.	S12, S24, S48
Psychosocial determinants of HAPA	Questions with a Likert-type scale	Questionnaires targeting motivational and volitional components of behavior change	Inclusion, M6, M12
