# Supplementary material for: Effectiveness and Implementation of Adapted Physical Activity Delivery Strategies for Older Adults Living With HIV in Ivory Coast: Protocol for a Type 2 Hybrid Randomized Controlled Trial
Source: JMIR Res Protoc. 2026 Jan 6;15:e84677. doi: 10.2196/84677 (PMC12820546; doi:10.2196/84677)
Supplement: Multimedia Appendix 3 [file resprot_v15i1e84677_app3.pdf]

ANRS - GRILLE D'EVALUATION *Projet Recherche*

Demandeur	Expert
M. DEBEAUDRAP Pierre	Rapporteur A

Titre : Faisabilité et efficacité d'un programme pilote d'activité physique sur les limitations fonctionnelles et le handicap associés au VIH chez les plus de 50 ans en Côte d'Ivoire.

PARTIE I : Evaluation Scientifique

- **Le projet relève-t-il des missions scientifiques de l'ANRS ?** **oui**
- **Qualité du projet (scientifique et technique)**

Pertinence pour la recherche sur l'infection à VIH, les hépatites virales et/ou co-infections	**Excellent**
Progrès par rapport à l'état actuel des connaissances	**Bon**
Définition des hypothèses et des objectifs	**Excellent**
Pertinence de l'approche méthodologique, statistique et/ou technologique	**Bon**
Impact potentiel du projet	**Excellent**

- **Faisabilité du projet**

La durée du projet est-elle raisonnable pour sa réalisation ?	**Bon**
Environnement scientifique et ressources du laboratoire (collaboration, missions/déplcaements, conditions de sécurité)	**Excellent**

PARTIE II : Adéquation budget / projet

- **Coûts**

Fonctionnement	**Raisonnable**
Equipement (< 16 000 euros HT)	**NonApplicable**
Personnel	**Raisonnable**
Participation demandée à l'ANRS	**Raisonnable**
Coût total estimé du projet	**Raisonnable**

PARTIE III : Dimension éthique

Les problèmes éthiques ont-ils été pris en considération (homme, animal) ?	**Oui**
Si le projet relève de la règlementation applicable à la recherche sur la personne, les demandes nécessaires ont-elles été prises en considération?	**Oui**

PARTIE IV : Respect de la charte d’éthique de la recherche dans les pays en développement

	**Ce sujet est-il abordé dans le projet ?**	**Si oui, de façon satisfaisante ?**
L’impact potentiel de la recherche pour la collectivité en terme de santé publique est-il envisagé ?	**Oui**	**Excellent**
Le rapport bénéfice-risque pour la personne participante est-il évalué ?	**Oui**	**Excellent**
Des moyens pour assurer la confidentialité sont-ils pris ?<br>(confidentialité liée à la séropositivité, aux données personnelles, …)	**Oui**	**Moyen**
Un médecin référent sera-t-il désigné pour chaque participant ?	**NonApplicable**	**NonApplicable**
La constitution d’un comité indépendant est-elle prévue ?	**Oui**	**Bon**
Des moyens pour éviter les conséquences discriminatoires ou stigmatisantes de la recherche sont-ils pris ?	**Oui**	**Moyen**
Un counselling pré et post test de dépistage est-il prévu ?	**NonApplicable**	**NonApplicable**
La prise en charge médicale pendant la recherche est-elle assurée ?<br>(par le projet, par le système de santé du pays, etc…)	**Oui**	**Excellent**
Les conditions de prise en charge post-recherche sont-elles définies ?	**Oui**	**Bon**
Des moyens pour communiquer les résultats de la recherche aux participants sont-ils définis ?	**Non**	**Insuffisant**
Les bénéfices de la recherche seront-ils rendus accessibles à la personne participante ?	**Non**	**Insuffisant**
Des représentants qualifiés de la communauté ou des associations de personnes vivant avec le VIH ou une hépatite virale sont-ils impliqués dans la mise en place et le déroulement de ce projet de recherche ?	**Oui**	**Moyen**
Si une notice d’information et/ou un formulaire de consentement sont fournis, leurs contenus vous paraissent-ils adaptés ?	**Oui**	**Excellent**

Faisabilité et efficacité d'un programme pilote d'activité physique sur les limitations fonctionnelles et le handicap associés au VIH chez les plus de 50 ans en Côte d'Ivoire.

Durée du projet : 36 Mois
Budget : 450 k
The overall objective of this research is to provide robust evidence on the clinical effectiveness and on potential implementation strategies for a physical activity (PA) intervention to improve the functional status and prevent disability among older people living with HIV from Ivory Coast.
The main specific objectives of this project are:
- To document the importance of HIV-related disabilities, notably the frequency / nature of functional limitations, neurocognitive impairments, and restrictions of social participation
- To evaluate the effectiveness of a structured PA program to the reduce functional limitations and disabilities
- To evaluate and compare different implementation strategies:
    - Success measured by the fidelity, coverage, and completeness
    - Identification of the barriers and facilitating factors
    - Perception and acceptability by patients

Research hypothesis
- Functional and activity limitations as well as restriction in social participation are frequent among PLWH and the frequency increases with age
- The practice of regular PA improves the functional performance (walking ability) and the strength, thereby reducing the risk of disabilities
- The practice of regular PA lower anxiety and depressive symptoms and has a positive impact on social participation and quality of life
- Home-based training can facilitate the regular practice of PA

Design
Implementation hybrid type 2 research to assess the clinical effectiveness and the implementation strategies of a physical activity intervention.
Participants will be randomized 1:1:1 to one for the following group: (1) intervention group with training sessions provided at the health center, (2) intervention group with home-based training and (3) control group. The control group will be offered counseling on physical activity and will have the opportunity to receive the intervention after completion of the M6 evaluation.
The study will be conducted in two primary health centers of Abobo, a popular area in the suburb of Abidjan.
It is planned to enroll 180 participants (60 per group)
Baseline assessment
Adults living with HIV of age ≥40 years who have been receiving ART for ≥12 months compared to HIV-uninfected participants of similar age and sex.
The main studies endpoints will be compared:
- The functional performances at the 6MWT, 5 sit and stand-up test (5SST), and grip strength
- Proportion of participants with and without HIV infection having Short Physical Performance Battery (SPPB) score < 10
-Proportion of participants with and without HIV infection having neurocognitive impairment and multivariate normative comparison of the cognitive scores
-The WHODAS-2, DFI and HDQ scores

Intervention assessment
Inclusion criteria: Age > 50 years, Participation in the baseline assessment, Presence of ≥1 criterion of functional limitation or disability: performance at the 6MWT or the 5 sit and stand up test (5SST) or grip strength below the 1st quartile, or WHODAS score ≥3

Non-inclusion criteria: pregnancy, acute OI, recent myocardial infection or unstable angina or unstable severe high blood pressure or unstable / severe respiratory disorder
Primary endpoint: difference in the 6MWT scores at 6-month post-intervention between groups.
Secondary endpoints: differences between groups at 6 and 12 months for
- The 5SST and grip strength tests
- The 6MWT scores (12-month post-intervention)
- The proportion of participants having SPPB score < 10 (and odds ratio) ;
- The proportion of participants having neurocognitive impairment
- The WHODAS-2, DFI and HDQ scores
- PHQ-9 scores and in the proportion of participants with a score ≥10
Implementation evaluated according to the RE-AIM model ("Reach Effectiveness Adoption Implementation Maintenance") with one table describing the methods for each outcome.
Intervention description
Structured program of physical activities including aerobic and strength exercises, with training sessions of 45 minutes over a 6 months period.
The training sessions will be organized on a weekly basis (2 per week?). Two implementation strategies will be proposed:
- First group (center-based intervention): all sessions will take place at the health center;
- Second group (home-based intervention): the first session(s) will be organized at the health center and the following sessions will take place at home but one session every month will be organized at the health center for supervision and re- assessment.
After completion of the M6 evaluation, participants of the intervention group will be offered to participate to the monthly supervision session (and to continue the training at home). Participants of the control group will be offered to receive the intervention and to choose the delivery modality (home-based or center-based).
Sample size: 180 participants (60 per group) will provide 80% power to show a significant difference between the center-based arm and the control arm with a type I risk set at 0.025 based on the results of the VIRAGE study (422 vs 380 meters for 6MWT score).

Analyse critique
Le projet est très bien écrit, très clair et porte par des équipes expérimentées sur cette thématique. Ils ont déjà montre dans l'étude VIRAGE que des PVVIH avec une activité physique importante avaient une meilleure performance au test de marche 6 minutes que ceux avec une faible activité physique alors que cette différence n'était pas significative chez des patients non-infectes par le VIH. La différence apparaissait après l'age de 50 ans.
On peut s'interroger du coup sur l'intérêt de répéter l'évaluation baseline mais cela va permettre de sélectionner les participants pour l'étude interventionnelle. Néanmoins, il me semblerait logique de mettre le même critère d'âge a 50 ans pour la baseline dans les critères d'inclusion. Il serait intéressant de stratifier également sur la durée d'infection VIH qui peut jouer un rôle dans le vieillissement accéléré.
Le projet se justifie pleinement au regard du vieillissement de la population VIH, du fait que deux méta-analyses ont montré le bénéfice d'une activité physique d'au moins 6 semaines chez les PVVIH sur la réduction du handicap et l'amélioration de la fonction cardio-respiratoire, la force, le poids et la qualité de vie mais que peu d'études ont été conduites en Afrique avec une composante de recherche implémentation.
Le choix des trois bras me semble justifie puisque l'on peut s'attendre à des différences en terme de niveau de formation et d'adhésion / observance enter les bras 1 et 2. L'adhésion et l'observance seront justement évaluées dans la partie implémentation du projet. Il n'est pas précisé ce qui sera fait en cas de non-respect des sessions de formation hebdomadaires pour le bras 1 et mensuel pour le bras 2.
En revanche, le switch a 6 mois des patients du groupe 1 au groupe 2 et du groupe 3 au groupe 1 ou 2 pose question quant à l'évaluation a M12 puisqu'il n'y aura plus de groupe contrôle. Il m'apparaitrait plus logique de poursuivre la comparabilité des groupes jusqu'à M12 ou si les bénéfices sont déjà clairement évidents à M6, de switcher tous les groupes en sessions à la maison après accord du SAB et de poursuivre l'évaluation sans comparaison de l'efficacité et en s'attachant plus aux paramètres d'implémentation comme la continuité ou l'acceptabilité de l'intervention.

Les critères de jugement sont clairs et cohérents mais seront difficilement comparables à M12 en l'absence de groupe contrôle et si la majorité des patients du groupe 3 choisissent d'appliquer l'intervention a la maison.
Il serait intéressant de compléter le bilan médical par une évaluation lipidique et hépatique (NASH) pour évaluer l'efficacité de l'intervention sur ces paramètres.
Le gros plus de cette étude est la prise en compte de nombreux paramètres d'implémentation qui me semble parfaitement cohérents et évaluables avec une approche collaborative qui devrait faciliter le transfert aux autorités nationales et renforcer les capacités sur cette thématique.
Les deux notes d'information sont claires et bien détaillées.

CONCLUSION

Projet innovant, bien construit qui devrait apporter des informations importantes pour diminuer le risque de handicaps chez les PVVIH de plus de 50 ans avec un transfert immédiat des résultats aux autorités nationales.

NOTATION GENERALE

AAvisPrioritaire
